# Digital Resources and Social Connectedness Among Ethnic Minority Older Adults: Systematic Review and Meta-Analysis

**DOI:** 10.2196/84962

**Published:** 2026-04-02

**Authors:** Padmore Adusei Amoah, Edward Kwabena Ameyaw, Miron Kumar Bhowmik, Prince Nyansah Adotey, Andrew Donkor

**Affiliations:** 1 School of Graduate Studies, Lingnan University, Hong Kong, 8 Castle Peak Rd, Tuen Mun, China (Hong Kong), 852 54876314; 2 Department of Education Policy and Leadership, The Education University of Hong Kong, Tai Po, China (Hong Kong); 3 Department of Medical Imaging, Faculty of Allied Health Sciences, Kwame Nkrumah University of Science and Technology, Kumasi, Ghana; 4 IMPACCT (Improving Palliative, Aged and Chronic Care through Clinical Research and Translation), Faculty of Health, University of Technology Sydney, Sydney, Australia

**Keywords:** digital resources, ethnic minority, older adults, social connectedness

## Abstract

**Background:**

Being socially connected is essential for health and well-being. Nonetheless, many older adults face social isolation, especially in ethnically diverse societies. Digital technologies offer a pragmatic approach to addressing problems with social connectedness; however, a consolidated understanding of their association with social connectedness among ethnic minority older adults remains unaddressed.

**Objective:**

We conducted a systematic review and meta-analysis to investigate the prevalence and utility of digital resources in promoting social connectedness among ethnic minority older adults.

**Methods:**

This systematic review and meta-analysis was guided by the PRISMA (Preferred Reporting Items for Systematic Reviews and Meta-Analyses) guidelines. Studies were retrieved from 4 electronic databases. A random-effects meta-analysis was used to estimate the pooled prevalence along with its 95% CI.

**Results:**

Twelve studies were included. The research approaches used by the included studies were as follows: quantitative (n=5), qualitative (n=5), and mixed methods (n=2). Overall, the estimated prevalence of internet use, which was defined as engaging in online activities through digital devices, was 81.8%. Commonly used digital devices were computers (66.7%), smartphones (63.2%), and tablets (29.3%). Sociodemographic, economic, and health-related factors influenced the adoption and use of digital resources among ethnic minority older adults. These older adults relied on digital resources to build meaningful social connections through social participation, maintaining cultural and religious ties, promoting transnational social networks, and fostering their potential for social connectedness. However, digital resources were not always useful for social connectedness and adversely affected in-person contact.

**Conclusions:**

Digital resources can promote social connectedness among ethnic minority older adults by enabling them to maintain their cultural and social values. Cities and regions aiming to promote the social connectedness and well-being of ethnic minority older adults should consider intersectional factors that affect access to digital resources and the sustainable adoption of digital resources.

## Introduction

There are approximately 5.6 billion internet users worldwide, which accounts for 67.9% of the global population, indicating the fundamental and crucial role of digital resources in the global socioeconomic and developmental milieu [1]. Digital resources encompass any electronic device, tool, system, or equipment that can connect to the internet and generate, store, and/or process data [2,3]. Digitalization and technological advancement are rapidly becoming the mainstay of contemporary communication, education, economic opportunities, and how people interact with their communities, even in low- and middle-income countries [4]. Digital engagements are increasingly underpinning most activities, and this situation also applies to ethnic minority older adults, especially given the myriad sociocultural barriers they face in accessing needed social services and maintaining healthy social connectedness [5]. Social connectedness, the primary focus of this study, is considered the antonym of loneliness as it involves constructive relationships with others, including groups, communities, and individuals [6]. Thus, it is strongly related to social connections or networks, which represent “the structure, function, and quality of our relationships with others” [7], including family, friends, neighbors, and people within and outside our immediate and external sociophysical environments [6,8]. Such connections are fundamental to individual and social sustainability, resilience, safety, and prosperity, especially among vulnerable people [9].

Older adults, especially those in minority groups, are often vulnerable to social isolation (ie, an object deficit in adequate social connections) and loneliness (ie, psychological pain due to the gap between desired and actual social connections) due to limited opportunities to be socially connected—a situation often compounded by language barriers and economic challenges [7,10,11], which can have a negative impact on their health [12]. It is estimated that one-third of older adults experience some degree of social isolation as their social networks reduce with age [13,14]. This has been attributed to factors such as migration, death, and the age-related functional decline of their usual social connections [10]. Acculturation is the psychological, social, and cultural changes that occur when 2 cultures come in contact [15]. Many migrants, especially those of ethnic minority status, tend to struggle to effectively participate in the traditions, values, and practices of dominant cultures, making them feel a sense of exclusion [16]. A major challenge is often how people from minority backgrounds decide whether to prioritize their heritage or the host culture [16]. Such experience leads to acculturative stress and psychological and emotional strain (eg, language barriers, feelings of being an outsider, experiences of discrimination, and even racism) as people adapt to a different culture [17]. Difficulty in acculturating to a new culture means many ethnic minority people face significant challenges forming meaningful relationships with members of the majority group (ie, social connectedness), something that is likely more abundant among majority populations [18,19]. Likewise, from the perspective of the social identity theory [20], such challenging experiences due to acculturation stress leave questions about how ethnic minority people form or maintain their self-concept relative to the larger population and culture in terms of how they categorize themselves (eg, in terms of race, nationality, and social class), how they identify with the group (ie, the majority group), and how they compare themselves with majority groups and those of similar characteristics as themselves (in-group) [21]. This process of constructing and maintaining social identity can be inimical to the forms of social connections and connectedness available to ethnic minority people, as the formation and maintenance of social relationships are also part of social identity [22].

These challenges with acculturation and social identity make digital tools and resources quintessential to the successful adaptation and social connectedness of ethnic minority people, particularly older adults, as they can provide them with space to reinforce their ethnic and cultural identities and connect with others with similar sociocultural backgrounds and beliefs [3,23]. Digital resources are broadly considered to comprise devices, such as smartphones, computers, tablets, and remote monitoring devices, and the platforms they enable, such as video streaming, messaging app use (eg, WhatsApp and WeChat), online gaming, and social media use (eg, Facebook, TikTok, and Instagram), through the internet [3]. Such resources can promote social connectedness among older adults and reduce loneliness and social isolation [24,25]. For ethnic minority older adults, being in unfamiliar and racially diverse communities reduces their social and cultural capital, leading to limited opportunity for them to generate meaningful social connections, which often affects their well-being adversely [18,26,27]. Despite the benefits and widespread use of digital resources, ethnic minority populations continue to face significant barriers to adopting and utilizing these resources [28]. Identified barriers include limited access to digital technology (eg, the internet), negative attitudes and technology anxiety, data privacy and security concerns, low digital skills, low literacy skills, and unfavorable device and content design [29,30]. These factors align with the Technology Acceptance Model, which argues that factors, including perceived ease of use, perceived usefulness of a technology, attitudes, and intention toward technology use, determine how much a person is willing to accept and adopt different technologies [31,32]. Given these factors, emergent questions include how and to what extent digital tools/platforms serve as mediums for social connections among ethnic minority older adults.

To address such research gaps, some systematic literature reviews have summarized studies on the impact of digital resources on social connectedness among older adults [24,33-36]. However, these studies have mainly focused on older adult populations, leaving a significant knowledge gap regarding specific groups, which complicates efforts to develop inclusive digital strategies, particularly those focused on ethnic minority aging populations, many of whom have difficulty forming social connections [10]. Therefore, this systematic review and meta-analysis seeks to identify the prevalence and utility of digital resources for social connectedness among ethnic minority older adults. The findings from this study can pave the way for well-situated future research and a strengthened knowledge base for responsive policies aimed at promoting social connectedness among ethnic minority older adults to prevent and manage ill-consequences such as social isolation and loneliness.

## Methods

### Overview

This systematic review was conducted and reported in accordance with the PRISMA (Preferred Reporting Items for Systematic Reviews and Meta-Analyses) guidelines [37]. The PRISMA checklist is provided in Checklist 1. It was registered with the International Prospective Register of Systematic Reviews (PROSPERO; CRD420251035114) [38].

### Study Selection Criteria

#### Participants

This systematic review included studies in which some or all study participants were ethnic minority older adults, as defined in various studies. An ethnic minority group is a group of people who share primarily racial and ethnic identities that differ from the dominant race in a given place. In most places, such people include immigrants (of different races/ethnicities and often different nationalities), indigenous people, people of color, and ethnic groups with characteristics such as different cultures/traditions and languages [27].

#### Intervention

Studies were included if they (1) explored the use of digital resources for communication or social function among ethnic minority older adults and (2) evaluated the association between the use of digital resources and outcomes related to social connectedness among ethnic minority older adults.

#### Studies

This systematic review and meta-analysis included primary studies of any design, as well as peer-reviewed journal articles and studies published in English. Editorials, opinion pieces, comments, letters, reviews, and any non–peer-reviewed materials were excluded.

#### Outcome Measures

The primary outcome of interest was the utility of digital resources for social connectedness. The secondary outcomes were the prevalence of digital resource usage and the factors that influence its use among ethnic minority older adults.

### Data Source and Search Strategy

The following electronic databases were searched: PubMed, Web of Science (Core Collection), PsycINFO, and Scopus. Databases were searched between May 2, 2025, and May 4, 2025. Consistent with our consideration of digital resources as electronic devices and their platforms used for social connections, the search strategy included use of a wide range of terms related to the following concepts: electronic devices and platforms (eg, computers, smartphones, tablets, and social media), social connectedness (with related terms such as social network, social connection, social support, social capital, and social interactions), ethnic minority populations (with related terms such as people of color, minority group, human migration, racial minority, aboriginal, and others), and older adults (with related words such as elderly, seniors, and older persons). Detailed information is provided in Table S1 in Multimedia Appendix 1. The expansive search terms enabled the retrieval of comprehensive results of related research. Medical Subject Headings (MeSH), keywords, and free-text terms were combined using “AND” or “OR” Boolean operators. A hand search through Google Scholar and tracing of reference lists of relevant studies were performed to supplement the electronic database search. No geographical restrictions were applied to the search.

### Study Selection

Duplicate articles were removed. Two reviewers independently screened the titles and abstracts of articles retrieved by the search to determine their eligibility. Agreement between reviewers during the title and abstract screening was almost perfect (Cohen κ=0.88). A third reviewer resolved any disagreement between the 2 reviewers. Full texts of any potentially relevant articles were obtained and assessed by 2 independent reviewers based on the defined inclusion criteria, with a Cohen κ score of 0.92. Disagreements were resolved by discussion with a third reviewer. The selection process was recorded in detail to produce a PRISMA flow diagram. EndNote (Clarivate) was used to manage the references.

### Data Extraction

For studies that fulfilled the inclusion criteria, 2 reviewers independently extracted relevant data. A standard data extraction form was developed. The following data were extracted:

- Study characteristics: authors, year of publication, country, aim, study design, inclusion and exclusion criteria, sample size, gender distribution, and mean (SD) age
- Outcomes/results: type of digital resource, prevalence data, and determinants of digital resource use
- Conclusions: author discussion/conclusions, limitations, funding, and notable conflicts of interest

### Study Quality Assessment

Two reviewers assessed the quality of the included studies. Any discrepancies were resolved by discussion. Interrater agreement between the reviewers was excellent, with a Cohen κ score of 0.90. Qualitative and quantitative studies were assessed by using the appropriate Joanna Briggs Institute (JBI) Critical Appraisal Checklist for qualitative research. Consistent with approaches reported in other previous systematic reviews and to facilitate comparison, each item in the appraisal checklist was rated as follows: yes (1 point), no (0 points), and unclear/not applicable (0 points) [39,40]. The sum was divided by the number of items in the appraisal checklist and multiplied by 100 to obtain a percentage. The risk of bias scores were categorized as follows: 80% or more (high quality), 50%‐79% (moderate quality), and below 50% (low quality), as suggested elsewhere [41].

Overall, most of the included studies fulfilled the relevant JBI criteria. Four quantitative studies were rated high quality [42-45]. Similarly, 4 qualitative studies were rated high quality [46-49]. Among the included studies, only 4 reported using a theoretical framework to guide their work [42-44,47].

Details of the quality assessment are provided in Tables S2 and S3 in Multimedia Appendix 1.

### Data Analysis

Data were summarized statistically when available, sufficiently similar, and of sufficient quality. The included studies were characterized by the study design and the type of digital resource. Qualitative findings were synthesized thematically following the procedure described by Thomas and Harden [50]. This included (1) free line-by-line coding of the findings of primary studies (eg, identifying prevalence indicators of digital resources and different uses of digital resources for social connections such as religious, cultural, intranational, and cross-national connections), (2) organization of codes into related categories to develop descriptive themes that capture patterns across the included studies (eg, religious and cultural codes were pulled together), and (3) development of higher-order analytical themes (eg, defining and refining categories and names to represent the core idea, such as maintaining cultural and religious connections). The analysis was mostly deductive, with a focus on addressing the research objectives guided by evidence from the literature on how digital resources affect social connectedness. Two authors led the data analysis, and the other members helped resolve contradictions during the process.

A meta-analysis was conducted on studies that reported quantitative data. A random-effects meta-analysis was used to calculate the pooled data with 95% CIs. The extracted data were used to compute the SE of the prevalence of digital resource usage for each included study, using the equation below, where n represents the sample size and p represents the sample proportion [51].


$$SE=\sqrt{\frac{p\times (1-p)}{n}}$$

Heterogeneity among studies was estimated using the *I*^2^ index [52], with values categorized as follows: 0%, no heterogeneity; 25%‐50%, low heterogeneity; 51%‐75%, moderate heterogeneity; and >75%, high heterogeneity. Heterogeneity was considered statistically significant at a *P* value of .05. Forest plots and tables were generated for the visualization of the results. Data were analyzed using Review Manager 5.4 (Cochrane).

## Results

### Search Results

**Figure 1.**
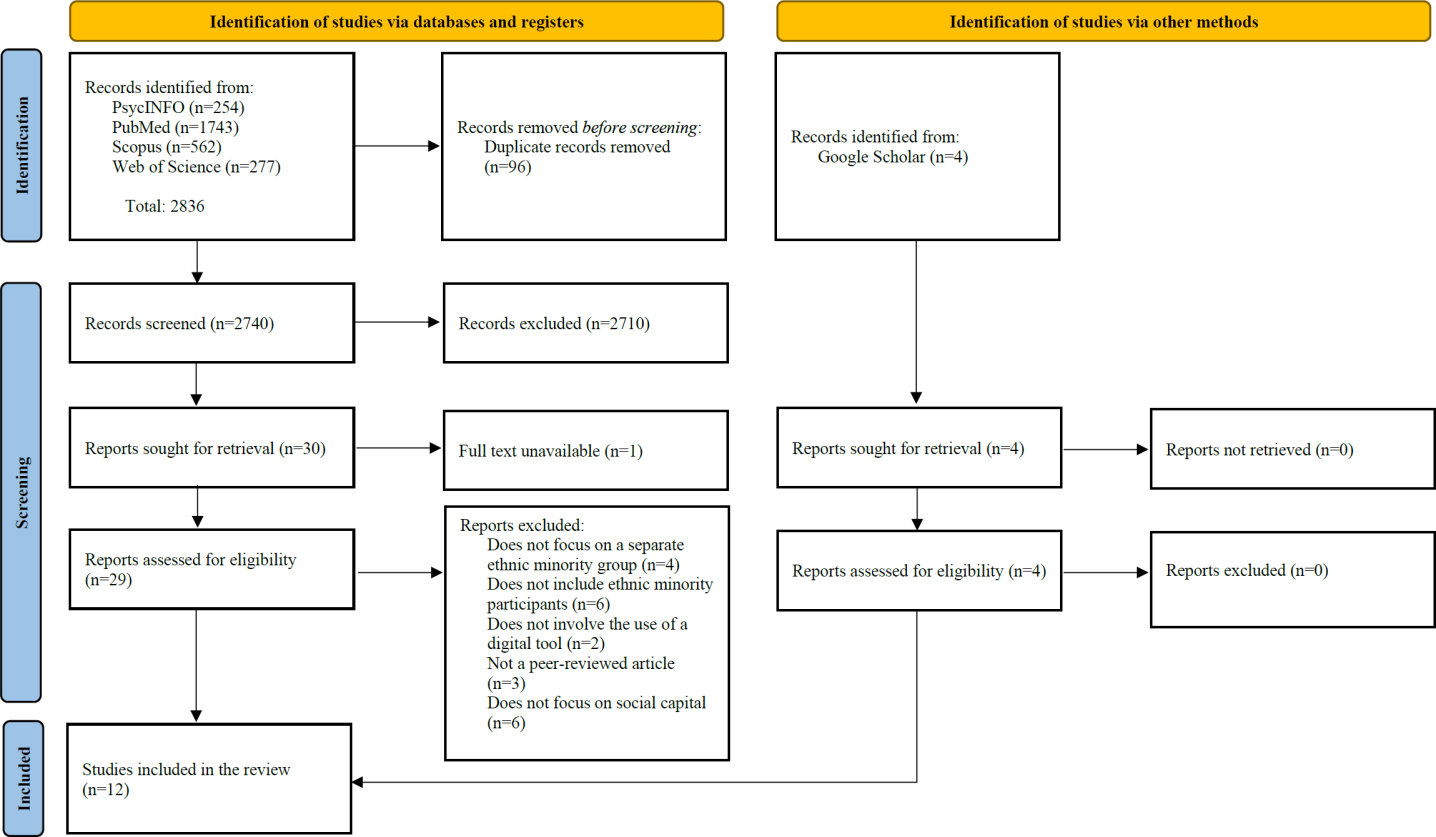
Screening of studies based on the PRISMA (Preferred Reporting Items for Systematic Reviews and Meta-Analyses) flow diagram [37].

An initial search of 4 electronic databases yielded 2836 articles, of which 96 duplicates were removed. The remaining articles were screened by title/abstract, and 2710 articles were excluded using the exclusion criteria. Of the remaining 30 articles, 8 met the inclusion criteria. Four additional articles were included through a Google Scholar hand search. Finally, 12 articles were included in this review (Figure 1).

### Characteristics of the Included Studies

Table 1 presents the characteristics of the included studies. The research approaches used by the included studies were as follows: quantitative (n=5) [42-45,53], qualitative (n=5) [46-49,54], and mixed methods (n=2) [55,56]. The included studies were mainly conducted in 4 central countries (United States, n=6; Australia, n=3; Finland, n=1; Israel, n=1), and 1 study was conducted in multiple countries.

**Table 1.** Characteristics of the included studies.

Study and country	Aim	Study design	Inclusion criteria	Type of digital resource	Dimensions of social connectedness	Sample size	Mean age (years)	Key results	Key arguments
DeLange Martinez et al [42], United States	To examine associations between PU^a^, PEOU^b^, ICT^c^ use, social connectedness, and loneliness among low-income, older Asian Americans during the COVID-19 pandemic	Cross-sectional	Older Asian Americans in affordable senior housing	ICT	Social integration and cultural connection	401	79.07 (SD 7.0)	PEOU was positively associated with PU (β=.15; *P*=.01). PEOU and PU were positive predictors of ICT use (PEOU: β=.26; *P*<.001; PU: β=.18; *P*=.01). ICT use increased social connectedness and reduced lonelines.	The COVID-19 pandemic spurred an exponential increase in the use of ICT for social connection. When ICTs are perceived as more useful, their usage tends to increase. Increased usage and familiarity make it easier, encouraging individuals to master new skills and diversify their usage.
Lee et al [56], United States	To explore whether a socially assistive robot (Hyodol) would have positive effects on Korean American immigrant older adults’ health behaviors and emotional well-being and whether the older adults would be receptive to the robot	Mixed methods	Being aged 65 years or older; Having the ability to engage in verbal conversations and understand either Korean or English; Having the willingness to take Hyodol (in a boy or girl costume) home and use it	Hyodol socially assistive robot	Social integration, and cultural and emotional connections	30	82.6 (SD 6.36)	Themes: (1) Bridging the gap in interpersonal relationships; (2) Serving individual health functions Barriers to adoptability: (1) Technological challenges; (2) Health-related and other challenges	Encouraging regular communication with robot companions in their mother tongue at home may help mitigate feelings of depression and loneliness among immigrant older adults by offering an avenue for social interaction.
Adeniji and Ashirifi [46], United States	To contribute to a broader understanding of the coping mechanisms employed by older African immigrants to promote social connections and interactions	Qualitative	Being aged 60 years or older; Having lived in the United States for 1 or more years; Being born in sub-Saharan Africa	Social media platform (Facebook and Instagram); Television (TV); Phone calls and video	Social integration, and intergenerational, religious, and transnational connections	11	72.18 (SD 5.23)	Positive self-talk and adaptation: “I have to cope with it” Technology/social media: “If I cannot interact physically outside, I go through social media/ watch TV” Intergenerational engagement through caregiving: “They [grandchildren] are my immediate constituency” Digging deep through faith: “Interactions have been mostly within the church”	Older African immigrants’ coping strategies can be enhanced through education that focuses on English language and technology classes. Spending time with grandchildren brings them happiness and a sense of connection, and helps them learn English.
Nguyen et al [47], Australia	To illustrate the ways ICTs facilitate the transnational life course transitions of Vietnamese migrant grandparents in Australia through life course digital learning	Qualitative	NR^d^	ICT	Cultural and transnational connections	22	72.8	Changes and constraints faced in transnational lives Digital kinning: Maintenance of transnational care and social support networks Digital homing: Development of migrant belongings and preservation of social and cultural identities	Not all grandparents are proficient with digital skills and knowledge before their migration. Digital citizenship, which is developed through the migration process as a part of life-course learning, can help older migrants cope with their increasingly transnational lives.
Pandya [45], United States	To examine the impact of an IMP^e^ on sociocultural adaptation, coping, and quality of life of South Asian older widows emigrating in later life to live with their adult children in the United States	Quasiexperimental active control	Having an inclination to engage; Having immigrated to the United States to live with adult children in the recent 2-4 months (in the year 2017); Self-reported fair comfort levels with digital technology and smartphone usage	Graphical user interface software designed for synchronous, online multimedia sessions	Cultural, religious, intergenerational, and transnational connections	172	NR	Compared with the online informative games program, results indicated that the IMP group exhibited greater adjustment (Cohen *d* range=0.55‐0.87; *P*≤.01). Older widows with higher formal education, from Hindu and Buddhist backgrounds, who are financially independent, have other kith and kin in the destination country, are in fair health, and have moved in with their daughters or live with their adult children who are alone or only with a partner, reported higher scores on intended outcomes. Immigrant adult child’s gender and family configuration were stronger predictors, and IMP lessons attended and self-practice mediated the relationship between significant sociodemographics and outcomes.	IMP is a meaningfully engaging intervention for late-life immigrant South Asian older widows, enabling adjustment in the new social and family contexts.
Pan et al [53], Belgium and Netherlands	To explore the consequences of the coronavirus pandemic for older Chinese migrants in Belgium and the Netherlands in terms of how increased low-person contact and decreased social participation affect loneliness and how they navigate those situations during the COVID-19 pandemic	Cross-sectional	Chinese migrants in Belgium and the Netherlands aged 50 years or older	Telephone or social media	Social participation and transnational connection	98	NR	Reduced social participation increased loneliness (EXP β=7.028; *P*=.039) Problem-focused coping strategies (measured as increased non–in-person contact via telephone or social media) and emotion-focused coping strategies (measured as increased distraction through increased participation in individual activities) were not found to protect against increased loneliness during the pandemic.	In-person social contact, for example, seems to have been replaced, or even supplemented, by increased non–in-person contact: 43.9% had increased non–in-person contact with their children and 81.6% with nonkin members.
Kouvonen et al [43], Finland	To examine the associations among depressive symptoms and SRH^f^ with different dimensions of DIT^g^ use in older migrants	Cross-sectional	NR	DIT	Social interaction, social participation, social support, and emotional and intergenerational connections	1082	63.2 (SD 8.4)	After adjusting for sociodemographic and socioeconomic factors, depressive symptoms (OR^h^ 2.68, 95% CI 1.37‐5.24; *P*=.004) and poor SRH (OR 7.90, 95% CI 1.88‐33.11; *P*=.005) were associated with a higher likelihood of not using the internet daily. Depressive symptoms (OR 1.88, 95% CI 1.06‐3.35; *P*=.03) and poor SRH (OR 5.05, 95% CI 1.58‐16.19; *P*=.006) also increased the likelihood of smartphone nonuse. Depressive symptoms were additionally associated with a lower likelihood of social media use, and poor SRH was associated with a lower likelihood of using the internet for messaging and calling.	Social media can become an increasingly important source of feelings of connectedness in older age, particularly when retirement and declining health limit other forms of social engagement. Technology usage can increase older migrants’ ability to maintain and expand their dispersed support networks [41]. However, mental health problems, such as depression, may also result in decreased initiative to engage in new activities.
Jun et al [44], United States	To understand the role of ICT use on social support and life satisfaction	Cross-sectional	NR	ICT: Emailing, smartphone usage, internet searching, and general social media usage	Social participation and social support	150	67.5 (SD 6.96)	Hierarchical multiple regression results indicated that a high level of smartphone use was significantly related to a high level of social support from families (β=0.05; *P*≤.05). High levels of smartphone use (β=0.05; *P*≤0.05) and social media use (β=0.06; *P*≤.05) were significantly related to a high level of social support from friends. A high level of social media use was significantly associated with higher life satisfaction among Korean immigrant elders (β=0.02; *P*≤.05).	Many Korean immigrant elders are strongly attached to their home country’s culture, which emphasises collectivism and interdependence. Social service practitioners and Korean community agencies working with Korean immigrant elders must recognize their clients’ capacity for technology use and encourage its use.
Juul et al [48], Australia	To investigate the role of “touchscreen technology” in facilitating increased physical activity and stimulating social interaction in RACFs^i^ in order to decrease social and physical inactivity	Qualitative	NR	Touchscreen technology (a large 165-cm interactive portable touch screen, with Windows software installed, on a mobile stand that could be rolled from room to room)	Social interaction, social participation, and cultural and emotional connections	In-depth face-to-face interviews=15; Observational session=around 16 per ward	NR	New technologies can enhance meaningful physical and social engagement beyond cultural and linguistic barriers. Technologies encourage social interactions and reduce boredom among residential care older adults. Effective use of technologies in social engagement in residential care requires well-trained and motivated staff.	The use of technology not only provides residents with instrumental and functional support, but it also has the potential to stimulate social interaction with each other and with staff, and can help transcend language barriers. If key social dimsnions are not well-considered, technologies can cause reduced human contact.
Millard et al [49], Australia	To understand the increasingly important role of digital citizenship (the ability to participate in society online) in supporting the well-being of aging migrants	Qualitative	NR	Digital citizenship (the ability to participate in society online)	Social interaction, social support, and cultural, intergenerational, and transnational connections	15	NR	Older people’s maintenance of support networks and social engagement, as well as their access to health care services, can be enhanced when they are motivated to increase their digital literacy (the ability to use the internet for information and communication) through appropriate educational, technological, and social support. This support was more effective when developed through social learning systems that create communities of practice. Improving digital literacy has significant implications for the well-being of older migrants, as it can enhance their ability to exchange emotional support across distances.	Older migrants want to connect with members of their support networks who are both local and distant, and both online and offline. Older migrants enjoy greater direct access to their social networks, information, services, and personal interests. Older migrants report a greater sense of autonomy and social participation as their digital literacy increases. Social learning settings functioning as communities of practice provide effective means for developing digital literacy and digital citizenship.
Andonian [55], United States	To explore the meanings, occupational engagement, and experiences associated with computer use	Mixed methods	Being aged 65 years or older; Living independently in the community; Having the ability to give informed consent; Having some exposure to computer use; Having no severe cognitive or mental health issues affecting the ability to participate	Computer use: Tablets; laptops; smartphones; and any computer-associated tasks, such as using the internet, downloading media, and creating documents	Social participation, social support, and cultural and emotional connections	9	NR	Engagement with digital resources led to: (1) Self-expression through self-directed engagement that reflects one’s interests, personal history, values, identity, and roles; (2) Freedom and appreciation of unrestricted access to instant and multiple sources of information; (3) Personal growth and feeling open to new experiences; (4) Strengthened relationships that provide social and emotional support; (5) Shift in worldview and perception of the world as larger yet more reachable; (6) Membership in the digital world is active participation in the digital realm.	Older adults discuss sharing their neighborhoods and architecture with friends from around the world through sharing photos. Immigrants value computer experiences that allow them to exercise self-control, express themselves, and pursue their interests and values through participation in communities across the nation and the world.
Khvorostianov et al [54], Israel	To explore how using the internet may facilitate coping with the challenges of immigration in later life, based on the case of older Jewish immigrants from the Former Soviet Union in Israel	Qualitative	Immigrants who are active internet users	Internet use	Professional and social networks, and intergenerational and transnational connections	32	76	Results indicated that internet usage by the study participants involved (1) managing health; (2) nurturing professional interests; (3) maintaining and extending social networks; (4) appreciating the past; and (5) enjoying leisure. Each usage seemed to preserve and even strengthen the participants’ self-worth, improving their quality of life.	Managing health online helps participants cope with aging despite lacking the host language skills. Internet usage is not only vital for the participants’ health but also provides significant personal empowerment.

a PU: perceived usefulness.

b PEOU: perceived ease of use.

c ICT: information and communication technology.

d NR: not reported.

e IMP: internet-based meditation program.

f SRH: self-rated health.

g DIT: digital information technology.

h OR: odds ratio.

i RACF: residential aged care facilities.

### Meta-Analysis

#### Prevalence of Digital Resource Usage Among Ethnic Minority Older Adults

##### Prevalence of Internet Use Among Ethnic Minority Older Adults

**Figure 2.**
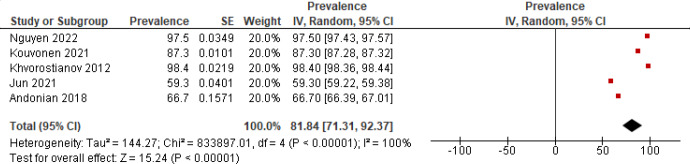
Forest plot of the prevalence of internet use [43,44,47,54,55].

Internet use was defined as engagement in online activities through digital devices. Five studies (1293 participants) reported prevalence data on internet use among ethnic minority older adults [43,44,47,54,55]. The estimated pooled prevalence of internet use was 81.84% (95% CI 71.31‐92.37). However, the observed between-study heterogeneity was significantly high (*I*^2^=100%; *P*<.001; Figure 2).

##### Overall Use of Information and Communication Technology

**Figure 3.**
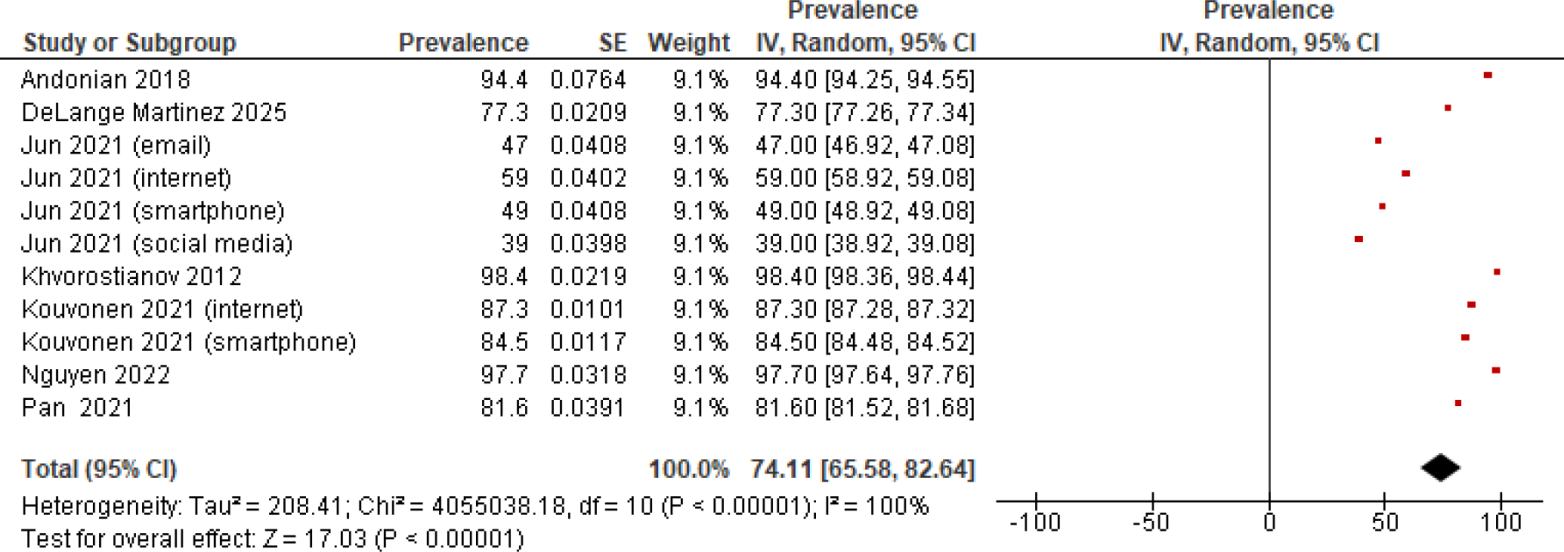
Forest plot of the prevalence of information and communication technology use [42-44,47,53-55].

The overall prevalence of information and communication technology resources that enabled digital engagements was calculated from 7 studies (1794 participants) [42-44,47,53-55]. The overall estimated pooled prevalence was 74.11% (95% CI 65.58‐82.64). However, a significantly high between-study heterogeneity was observed (*I*^2^=100%; *P*<.001; Figure 3).

##### Prevalence of Smartphone Use Among Ethnic Minority Older Adults

**Figure 4.**
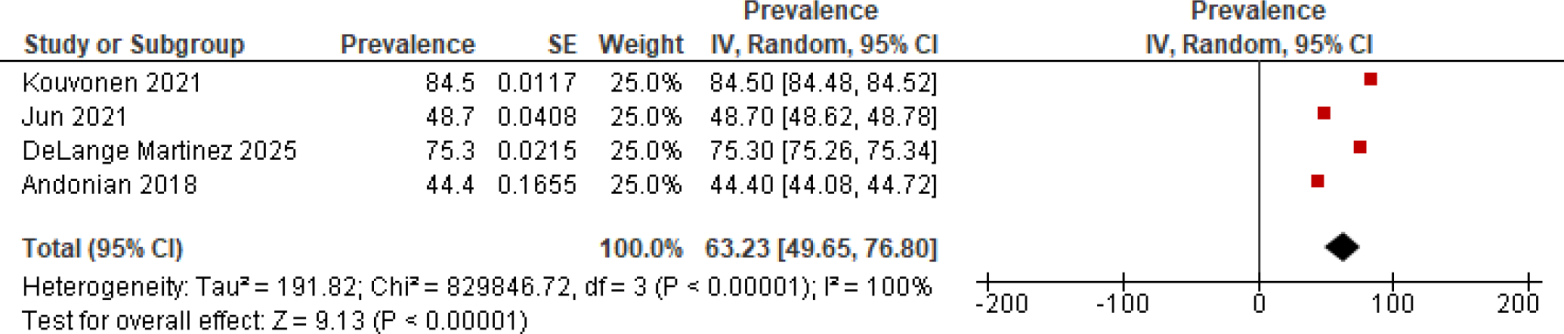
Forest plot of the prevalence of smartphone use [42-44,55].

Four studies (1525 participants) reported prevalence data on smartphone use among ethnic minority older adults [42-44,55]. The estimated pooled prevalence of smartphone use was 63.23% (95% CI 49.65‐76.80). However, a significantly high between-study heterogeneity was observed (*I*^2^=100%; *P*<.001; Figure 4).

##### Prevalence of Computer Use Among Ethnic Minority Older Adults

**Figure 5.**
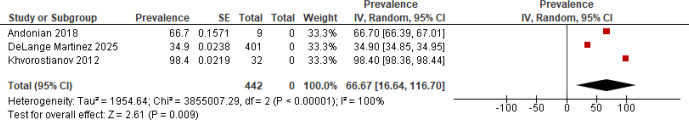
Forest plot of the prevalence of computer use [42,54,55].

Three studies (442 participants) reported prevalence data on computer use (ie, desktops and laptops) among ethnic minority older adults [42,54,55]. The estimated pooled prevalence of computer use was 66.67% (95% CI 16.64‐116.70). However, a significantly high between-study heterogeneity was observed (*I*^2^=100%; *P*<.001; Figure 5).

##### Prevalence of Tablet Use Among Ethnic Minority Older Adults

**Figure 6.**
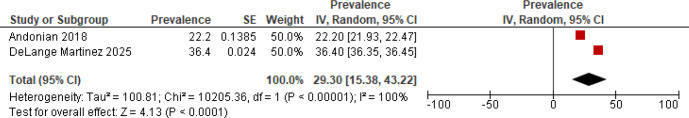
Forest plot of the prevalence of tablet use [42,55].

Two studies (410 participants) reported prevalence data on tablet use among ethnic minority older adults [42,55]. The estimated pooled prevalence of tablet use was 29.30% (95% CI 15.38‐43.22). A significantly high between-study heterogeneity was observed (*I*^2^=100%; *P*<.001; Figure 6).

### Thematic Synthesis

Thematic synthesis of the extracted data generated the following two major themes: (1) utility of digital resources for social connectedness, and (2) factors that influence the use of digital resources among ethnic minority older adults.

#### Utility of Digital Resources for Social Connectedness

##### Overview

The following six subthemes relating to the utility of digital resources for social connectedness were identified: (1) enhancing social integration and participation, (2) maintaining cultural and religious connections, (3) promoting transnational connectedness, (4) fostering intergenerational connectedness, (5) expanding social networks for social connectedness, and (6) downsides of digital resources for social connectedness.

##### Enhancing Social Integration and Participation

Findings related to digital resources enhancing social integration and participation emerged from quantitative (n=4) [42-44,53], qualitative (n=3) [46,48,49], and mixed methods (n=2) [55,56] studies. It was revealed that platforms, such as social media, enabled ethnic minority older adults to actively participate in social activities, access information and community resources, learn new skills, and interact with community members. For example, a cross-sectional survey of 98 Chinese immigrants in Belgium and the Netherlands found that 81.6% of respondents had more frequent non–in-person contact with kin and nonkin individuals via smartphone-enabled social media to support social participation during the COVID-19 pandemic [53]. Qualitative evidence highlighted the critical role of digital resources in promoting a sense of community belonging among ethnic minority older adults. A study among older adults from non–English-speaking backgrounds living in a residential aged care facility in Western Australia made the following argument [48]:

*[Touchscreen technology] lifted the mood of the room, transforming it into a livelier space and facilitating a sense of community and shared fellowship among all participants… residents went from being individuals doing nothing to active members of a group, contributing to a sense of belonging.*

##### Maintaining Cultural and Religious Connections

Evidence on digital resources playing a critical role in maintaining cultural and religious connections among immigrant older adults was drawn from 4 qualitative studies [46-49], 2 quantitative studies [42,45], and 2 mixed methods studies [55,56]. A quasiexperimental study showed that participants who received an internet-based meditation program across various devices demonstrated significantly greater sociocultural adaptation compared with a control group, with effect sizes ranging from 0.55 to 0.87 (*P*≤.01) [45]. It was observed that digital resources created a convenient virtual space for religious practices, particularly during the COVID-19 pandemic, by connecting the older adults with people of similar faith. In a study of 11 older African immigrants, it was reported that avenues, such as Facebook, created opportunities for social, cultural, and spiritual engagement with others during the pandemic [45]. For instance, a study among older immigrants in Western Australia made the following statement regarding individuals’ motivation for participating in the Internet Café community of practice [49]:

*…desire to connect with the places and memories of their past…on one occasion, the cafe was filled with Irish folk music as a patron played the traditional sounds of his homeland from a website.*

##### Promoting Transnational Connectedness

Seven studies (4 qualitative studies and 3 quantitative studies) indicated that the availability of digital resources, such as the internet, smartphones, and media, such as video calls or group chats, allowed immigrant older adults to maintain connections with family members and friends across countries [42,45-47,49,53,54]. A cross-sectional survey of 401 older Asian Americans found that 89.2% of respondents agreed that digital resources were useful for engaging family and friends [42]. Khvorostianov et al [54] noted that transnational connections formed through digital resources often led to empowerment and better integration of immigrants into their host societies, including the development of lasting social relationships. It was observed that digital resources played a critical role in facilitating financial transactions, thereby enabling stronger social connectedness through the continuity of transnational caregiving and support. For example, a participant in a qualitative study involving 22 Vietnamese immigrant grandparents made the following statement [47]:

*[She] maintained daily contact via video phone calls…with her son and grandchildren, as well as a group of Vietnamese close friends…For her mother, she provided transnational care by sending remittances to pay the domestic worker, cover her mother’s daily expenses, and purchase nutritional supplements.*

##### Fostering Intergenerational Connectedness

Findings from 3 qualitative studies [46,49,54] and 2 quantitative studies [43,45] indicated that digital resources fostered intergenerational connections among ethnic minority older adults. Digital resources were important interventions for bonding with grandchildren, facilitating intergenerational language exchange, sharing family history, and mentoring the younger generation. According to Kouvonen et al [43], the frequent use of social media among 631 older immigrants enabled intergenerational interactions. Khvorostianov et al [54] identified that the internet served as a tool that connected grandchildren to their heritage:

*[We] built a website where we publish articles, memories and documents telling how our relatives were murdered. My grandchildren browse this website too and learn about their family history.*

##### Expanding Social Networks for Social Connectedness

Evidence from 2 qualitative studies [49,54], 2 quantitative studies [43,44], and 1 mixed methods study [54] indicated that digital resources are critical for expanding the social networks of older adult immigrants. It was reported that internet-enabled computer usage helped ethnic minority older adults engage in social activities that expanded their social participation beyond their immediate environment [55]. Similarly, the use of social media platforms, facilitated by digital tools, supported development and reconnection with past social networks [43,44,49,54,55]. It was revealed that maintaining social networks online helped older adult immigrants cope with distance from family members and friends; thus, it provided physical and emotional support to remain socially connected and alleviate feelings of social isolation [49,54,55]. A study from Australia made the following statement [49]:

*Improving digital literacy has special implications for the well-being of older migrants because it can enhance their ability to exchange emotional support across distance.*

##### Downsides of Digital Resources for Social Connectedness

Notwithstanding, having access to digital resources was not always impactful in reducing the outcomes of social connectedness, such as loneliness [53]. Other studies indicated the likelihood that digital resources will reduce human contact, which can worsen quality of life [48]. In the article by Andonian [55], some participants presented instances that can lead to poor social connectedness through physical isolation, such as the following:

*When I came here, I didn’t know anything. Everybody was like running all over the city with, you know [points to smartphone]. What are they doing?! You know, they’re not saying “hello” to anybody around them…*

These observations align with others that indicate the limitations of digital resources in promoting social connectedness. For instance, it was found that having a poor health status and being socioeconomically disadvantaged led to less engagement with digital resources, making them less relevant to promoting low social connectedness, as presented in other findings [43,55].

### Factors That Influence the Use of Digital Resources Among Ethnic Minority Older Adults

#### Overview

The systematic review identified sociodemographic, economic, and health-related factors that influence the adoption and use of digital resources among ethnic minority older adults.

#### Sociodemographic and Economic Factors

##### Age

Older age was found to be negatively associated with digital resource use [42,43,46,47,49,54,55]. For instance, Kouvonen et al [43] reported that nonuse of the internet and smartphones was prevalent among individuals aged 65 years or older compared to younger age groups (internet nonuse: 97/421, 23.0% vs 40/646, 6.2%; smartphone nonuse: 98/351, 27.9% vs 51/614, 8.3%). Additionally, Nguyen et al [47] found that age was associated with low digital literacy among ethnic minority older adults (Vietnamese Australians). However, they were motivated to learn how to use digital resources due to the social connection opportunities they offered, such as participation in religious events.

##### Gender

Gender was found to be a predictor of digital resource use among ethnic minority older adults [42,49,54]. DeLange Martinez et al [42] revealed that female gender was significantly and negatively associated with the use of digital resources and platforms (r=–0.15; *P*=.01). Khvorostianov et al [54] found that older women were more likely to use the internet to develop new professional interests, whereas older men used it to maintain their original professional identity, which helped build their self-confidence as active members of their communities.

##### Educational Level

Seven studies found that ethnic minority older adults’ adoption and use of digital resources were influenced by their levels of education [42-47,54]. DeLange Martinez et al [42] found that older adults with higher educational levels were more likely to utilize digital tools for social connections compared to those with lower educational levels (β=.21; *P*<.001). Nguyen et al [47] stated that Vietnamese migrant grandparents with lower education were less likely to engage with digital resources, with most using them for the first time after their migration. To address disparities in the adoption and use of digital resources, instructions for these resources should be clear and simplified for ethnic minority older adults with limited educational backgrounds to ensure ease of use [45].

##### Language Proficiency

Few studies stated that language proficiency was related to the adoption and perceived ease of use of digital resources among ethnic minority older adults [42,43]. Kouvonen et al [43] reported that older Russian-speaking immigrants in Finland who had effectively integrated into Finnish society and had strong local language skills were more likely to own a smartphone to access the internet. Linguistic bias in digital resource and application development, which favors English-speaking users, needs to be addressed to facilitate the use of technology among culturally and linguistically diverse older adults [42].

##### Religion

One study found religion to be associated with the use of digital resources among immigrant South Asian older widows [45]. Pandya [45] identified that an internet-based meditation program was more effective for older Hindu and Buddhist widows than Christian widows because of cultural familiarity with meditation practices.

##### Economic Status

Economic status was identified as an influential factor in the digital technology used by ethnic minority older adults [43-45,49]. For example, Jun et al [44] reported that Korean immigrant older adults with an annual income greater than US $20,000 were significantly more likely to use digital resources than those in the lower-income group (*t*_148_=−4.53; *P*≤.001). The financial cost of digital resources, such as purchasing a smartphone or the monthly cost of internet services, was a significant barrier for low-income older adults, which increased the prevalence of nonuse of digital resources [43].

### Health-Related Factors

#### Physical Health

The physical health of ethnic minority older adults has been identified as a critical factor in the adoption and use of digital resources [43-45,55,56]. Physical health challenges, including decreased vision and coordination, affected the use of digital resources. For example, Kouvonen et al [43] reported that older Russian-speaking immigrants in Finland with poor physical health were 5 times less likely to own and use smartphones compared to those with good physical health (odds ratio [OR] 0.20, 95% CI 0.06‐0.69; *P*=.006).

#### Mental Well-Being

Mental health challenges, such as cognitive decline, anxiety, and depression, were identified as critical barriers to the adoption and use of digital resources among ethnic minority older adults [42,43,56]. As observed by Kouvonen et al [43], ethnic minority older adults experiencing depressive symptoms were almost 3 times more likely not to use the internet for messaging or calling compared to those without depressive symptoms (OR 2.68, 95% CI 1.37‐5.24; *P*=.004).

#### COVID-19 Restrictions

One study investigated the impact of the COVID-19 pandemic on the adoption and use of digital resources among ethnic minority older adults [53]. Pan et al [53] stated that COVID-19 accelerated the adoption and use of digital resources among older Chinese migrants in Belgium and the Netherlands, with increases of 43.9% and 81.6% in non–in-person contact with family and friends, respectively.

## Discussion

### Principal Findings and Comparison With Other Studies

Maintaining active social connections is critical to the health and well-being of ethnic minority older adults, as it reduces the risk of social isolation and loneliness [10]. Thus, understanding and promoting the dynamics of social connections among this group of people are important for protecting their health and well-being. The study found significant variation in the prevalence estimates of digital resource use among ethnic minority older adults, with most preferring to use the internet for social connectivity. Overall, the prevalence of information communication technology use was 74.11%, and the estimated prevalence of internet use was 81.8%. Moreover, the values for computers, smartphones, and tablets were 66.7%, 63.2%, and 29.3%, respectively. Internet use ranged from 59.3% among older Korean immigrants in the United States [42] to 98.4% among older Jewish immigrants from the Former Soviet Union in Israel [54]. These findings indicate that while digital engagement for social connection is considerable among ethnic minority older adults, usage disparities exist across ethnic groups [42]. In comparison to studies about older adults generally, which also note a digital divide, these results emphasize the critical role of ethnic and cultural context in shaping digital resource utilization [25,32]. The high prevalence of internet use suggests that targeted digital inclusion strategies could be highly effective. However, these must be culturally sensitive and address the unique needs and preferences of each ethnic minority group [28,42].

The qualitative synthesis identified two related themes: (1) the utility of digital resources for social connectedness, and (2) the factors that influence their use among ethnic minority older adults. These themes offer insights into the lived experiences of ethnic minority older adults in engaging with digital resources. It was revealed that digital resources function as essential tools for enhancing social integration and participation, maintaining cultural and religious connectedness, promoting transnational connections, and fostering intergenerational connectedness through expanded social networks. These findings suggest that digital resources facilitate access to information and opportunities for ethnic minority older adults to maintain meaningful social relationships with their families who have been left behind in their home countries or have been apart from them while also enabling these older adults to create new social connections to support their well-being [34,36]. From the perspective of social identity theory, digital resources can be instrumental in how ethnic minority populations construct and maintain their self-concepts [21,23]. In this review, such identity construction and maintenance are influenced by digital resources, which bridge geographical boundaries for social connectedness and enable ethnic minority older adults to meet cultural expectations, such as supporting younger generations.

Despite the overall positive aspects of digital resources for social connectedness, this review also points to instances or conditions that make them less relevant or even a causal mechanism for low social connectedness among aging ethnic minority people [53,55]. This finding underscores the importance of physical social connections in fostering social connectedness, as virtual interactions have significant limitations (eg, difficulty in conveying emotions effectively) [56]. These observations mean that the integration of digital resources in promoting social connectedness must be done from a balanced perspective—one that seeks to complement physical interactions with digital resources instead of attempting to entirely replace or overly prioritize the technologies over actual human contact [57]. For ethnic minority older adults, such a balance can be crucial to their acculturation, as found in studies showing that the high prevalence of smartphone use made entry into local cultural dynamics challenging for minority migrant individuals [55,56]. These downsides to digital resources were strongly related to intersectional factors, such as economic status, health status, and others, which require serious attention to enable the sustainable use of technologies in promoting social connectedness, as further elaborated in the following paragraphs.

The systematic review identified several factors that influenced the adoption and use of digital resources among ethnic minority older adults, which were categorized into sociodemographic, economic, and health-related factors. Identified sociodemographic factors included age, gender, educational level, language proficiency, and religion. These factors indicate the intersectional nature of digital engagement among older adults in general, with significant stratification across gender, race/ethnicity, education, and other areas [58]. Considering that many ethnic minority older adults are illiterate (at least in the context of host societies) and have poor language proficiency [26], their use of digital platforms/resources may be weaker than that of ethnic majority older adults in a given place, leading to fewer opportunities to build social connections. At the same time, limitations in language and other intersectional factors can also drive ethnic minority older adults to resort to digital resources [23]. In addition, the results across 7 studies were consistent on the issue that younger ethnic minority individuals were more likely to adopt and use digital resources, which further indicates the significant role of factors, such as educational attainment (mostly higher among young people), in the use of digital resources [42,43,46,47,49,54,55]. However, there was evidence that many ethnic minority older adults were highly motivated to utilize digital resources to stay connected with family and friends [46,47,55], which demonstrates the utility of digital resources for sociocultural adaptation and maintenance of social identity. A previous study has shown that the relationship between age and the adoption of digital resources is mediated by computer self-efficacy, cognitive ability, and computer anxiety [59]. Strong motivation toward digital inclusion can be crucial in cultivating and achieving desirable outcomes that promote social connection through digital means [5].

The review found that the income level of ethnic minority older adults significantly influenced their adoption and use of digital resources. A combination of factors, including high-cost digital resources, limited internet access, and a lack of digital skills, is a key barrier that excludes low-income older adults from accessing and using digital resources. This finding reinforces the theoretical perspectives of the fundamental cause theory, which argues that the health and well-being outcomes of populations are influenced by their access to critical socioeconomic resources [60]. It has been recommended that for ethnic minority older adults to experience the social connection benefits of digital resources, policies and programs cannot solely focus on the technological aspects of their digital inclusion, as economic resources are key to making such programs functional [49].

Furthermore, health-related factors significantly affected the adoption and use of digital resources among ethnic minority older adults. It was recognized that ethnic minority older adults experiencing physical or mental health challenges, such as cognitive decline, depression, anxiety, decreased vision, and coordination, were less likely to own and use digital resources. This is likely because physical and mental health issues can cause disinterest in activities of daily living, in addition to potential compromises to bodily functions needed for digital engagement, such as vision loss [25,28]. Going forward, a deeper understanding of these influential factors is essential for designing and implementing health-appropriate strategies that enable equitable access to digital resources among ethnic minority older adults. Moreover, as with other population groups [61,62], COVID-19 pandemic restrictions, including social distancing measures, led to an increased use of digital resources. Other studies among ethnic minority older adults have found similar results, showing that people used video calls to maintain social connections [63,64]. Thus, experiences during the pandemic can be instructive for incorporating digital tools to promote social connectedness among minoritized older adults whose sociocultural and adaptation issues weaken their opportunities for lasting social connectedness. Considering the dangers of social isolation as an “epidemic,” as observed during the COVID-19 pandemic, can hasten efforts toward integrating vulnerable older adults into social dynamics through digitalization.

### Strengths and Limitations

To the best of our knowledge, this is one of the first systematic reviews and meta-analyses to explore the adoption of digital resources to address social connection challenges and the factors influencing their adoption and use among ethnic minority older adults globally. The study followed a rigorous approach to searching relevant articles and extracting and handling data. However, the study has limitations that must be acknowledged when interpreting the findings. First, we limited data extraction to full articles written in English and with full text available, which may have excluded relevant studies. Second, the included studies used different age thresholds to define older adults, including one that recruited participants aged 50 years or older [53]. Divergence in age criteria means that the experiences of older adults across studies are likely to differ, potentially affecting the compatibility of results across the included papers. Third, substantial heterogeneity was detected across the included studies; hence, a random-effects model was applied. Fourth, the lack of information regarding the use of validated instruments in the quantitative studies may have introduced measurement bias. Additionally, it was not possible to infer causal relationships between the use of digital resources and social connectedness because almost all the quantitative studies used cross-sectional designs. Notwithstanding these limitations, this study offers rare, collated evidence on the characteristics and influence of digital resource use among the understudied group of ethnic minority older adults. The evidence offers an opportunity to conduct further empirical studies to better understand ethnic minority older adults’ engagement with digital resources and the causal pathways involved.

### Conclusion

The findings of this review suggest that digital resources can enhance social connections among older adults from ethnic minority groups. Intersectional factors related to sociodemographic, economic, and health-related aspects are influential in the adoption and use of digital resources by ethnic minority older adults to maintain social connections. Ethnic minority older adults utilize a wide range of digital tools/platforms to enhance their social integration in new or foreign societies, maintain their cultural and religious heritage, and foster intergenerational connections. The findings suggest that access to culturally appropriate and suitable digital resources for ethnic minority older adults with diverse health statuses can enhance social connectedness. While digital resources appear to be positively associated with social connectedness among ethnic minority older adults, future research can extend existing knowledge by examining how these resources may have negative consequences for the extent and nature of the social connections that older ethnic minority people secure. This is in view of some observations that digital resources can discourage in-person contact and may not necessarily be useful for addressing concerns with low social connectedness.

Additionally, it will be instructive to critically examine the diverse social connections formed by these older adults. This is because the types of social networks one possesses determine the nature of the resources embedded in those networks and the impact they will have on various life areas [65]. Moreover, research is needed on how ethnic minority older adults sustain the social connections they form through digital resources compared to those made in person, to advance social sustainability in aging through digital resources. At the same time, in recognition of the limitations and other factors that affect the impact of digital resources on social connectedness in aging societies, research is needed to better understand how various social technologies can be engaged sustainably (eg, effective use over the long term) among aging populations in different places. Finally, longitudinal study designs will be an ideal step toward understanding the stability in the relations between digital resources and social connectedness among older ethnic minority populations.

## Supplementary material

10.2196/84962 Multimedia Appendix 1 Search strategy and quality assessments.

10.2196/84962 Checklist 1 PRISMA checklist.

## Abbreviations

JBI: Joanna Briggs Institute
OR: odds ratio
PRISMA: Preferred Reporting Items for Systematic Reviews and Meta-Analyses
