# Supplementary material for: Digital Resources and Social Connectedness Among Ethnic Minority Older Adults: Systematic Review and Meta-Analysis
Source: JMIR Aging. 2026 Apr 2;9:e84962. doi: 10.2196/84962 (PMC13046222; doi:10.2196/84962)
Supplement: Multimedia Appendix 1 [file aging-v9-e84962-s001.docx]

**Multimedia Appendix 1**

**Table S1.** Search strategy.

| **PubMed 4^th^ May 2025**  1. “Aged” [Mesh] 3,688,546  2. "Frail Elderly"[Majr] 9399  3. "older adult*"[Title/Abstract] OR elder*[Title/Abstract] OR "older popula*"[Title/Abstract] OR "old people"[Title/Abstract] OR "older people"[Title/Abstract] OR "old person"[Title/Abstract] OR "older person*"[Title/Abstract] OR senior*[Title/Abstract] OR "frail elder*"[Title/Abstract] OR "functionally impaired elder*"[Title/Abstract] OR "functionally impaired elder*"[Title/Abstract] OR "frail older adult*"[Title/Abstract] OR geriatri*[Title/Abstract] OR "aging popula*"[Title/Abstract] OR aged*[Title/Abstract] OR "older individual*"[Title/Abstract] OR "old individual*"[Title/Abstract] OR "older age"[Title/Abstract] OR older-age[Title/Abstract] OR "old age"[Title/Abstract] OR old-age[Title/Abstract] OR adult*[Title/Abstract] 2851842  4. #1 or #2 or #3 5750987  5. "Minority Groups"[Majr] 9679  6. "Cultural Diversity"[Majr] 7884  7. "Cultural Deprivation"[Majr] 558  8. "Ethnic and Racial Minorities"[Mesh] 1212  9. "Human Migration"[Mesh] 28420  10. "Refugees"[Mesh] 14659  11. "Population Groups"[Mesh] 357818  12. "Anthropology, Cultural"[Majr] 96329  13. "Vulnerable Populations"[Majr] 6020  14. "Multilingualism"[Majr] 5266  15. "Ethnic and Racial Minorities"[Majr] 258  16. "Emigration and Immigration"[Majr] 15875  17. "aborigin"[Title/Abstract] OR "minority group"[Title/Abstract] OR "ethnic group"[Title/Abstract] OR "immigrant"[Title/Abstract] OR "refugee"[Title/Abstract] OR "ethnic minority"[Title/Abstract] OR "racial group"[Title/Abstract] OR "indigen"[Title/Abstract] OR "racial and ethnic diversity"[Title/Abstract] OR "multiracial"[Title/Abstract] OR "multicultural"[Title/Abstract] OR "racial and ethnic group"[Title/Abstract] OR "asylum"[Title/Abstract] OR "multilingual"[Title/Abstract] OR "vulnerable population"[Title/Abstract] OR "vulnerable community"[Title/Abstract] OR "vulnerable group"[Title/Abstract] OR "vulnerable neighborhood"[Title/Abstract] OR "low-income community"[Title/Abstract] OR "low income group"[Title/Abstract] OR "low-income population"[Title/Abstract] OR "marginalized community"[Title/Abstract] OR "marginalized group"[Title/Abstract] OR "marginalized population"[Title/Abstract] OR "marginalized neighborhood"[Title/Abstract] OR "underserved community"[Title/Abstract] OR "underserved group"[Title/Abstract] OR "underserved population"[Title/Abstract] OR "underserved neighborhood"[Title/Abstract] OR "disadvantaged community"[Title/Abstract] OR "disadvantaged group"[Title/Abstract] OR "disadvantaged population"[Title/Abstract] OR "disadvantaged neighborhood"[Title/Abstract] OR "black, indigenous and people of color"[Title/Abstract] OR "bipoc"[Title/Abstract] OR "people of color"[Title/Abstract] OR "immigrant"[Title/Abstract] OR "migrant"[Title/Abstract] OR "refugee"[Title/Abstract] OR "cald"[Title/Abstract] 102119  18. #5 or #6 or #7 or #8 or #9 or #10 or #11 or #12 or #13 or #14 or #15 or #16 or #17 549587  19. "Cell Phone"[Mesh] 25764  20. "Smartphone"[Mesh] 11534  21. "Computers, Handheld"[Majr] 9153  22. "Telemedicine"[Majr] 44240  23. "Webcasts as Topic"[Majr] 327  24. "Text Messaging"[Majr] 3906  25. "Mobile Applications"[Majr] 12009  26. "Medical Informatics Applications"[Majr] 231989  27. "Computer-Assisted Instruction"[Majr] 9826  28. "MP3-Player"[Majr] 146  29. "Telenursing"[Majr] 248  30. "Reminder Systems"[Majr] 2365  31. "smartphone"[Title/Abstract] OR "mobile telephone"[Title/Abstract] OR "cellphone"[Title/Abstract] OR "mobile"[Title/Abstract] OR "phone"[Title/Abstract] OR "i-pad"[Title/Abstract] OR "i-pod"[Title/Abstract] OR "i-phone"[Title/Abstract] OR "android"[Title/Abstract] OR "MP3 player"[Title/Abstract] OR "MP4 player"[Title/Abstract] OR "tablet"[Title/Abstract] OR "pda"[Title/Abstract] OR "personal digital assistant"[Title/Abstract] OR "handheld"[Title/Abstract] OR "smartwatch"[Title/Abstract] OR "wearable"[Title/Abstract] OR "mobile device"[Title/Abstract] OR "m-health"[Title/Abstract] OR "mobile health"[Title/Abstract] OR "e-health"[Title/Abstract] OR "electronic health"[Title/Abstract] OR "digital health"[Title/Abstract] OR "tele-medicine"[Title/Abstract] OR "telehealth"[Title/Abstract] OR "telecare"[Title/Abstract] OR "tele-nurse"[Title/Abstract] OR "tele-psychiatry"[Title/Abstract] OR "tele-monitor"[Title/Abstract] OR "tele-consult"[Title/Abstract] OR "tele-counsel"[Title/Abstract] OR "tele-coach"[Title/Abstract] OR "instagram"[Title/Abstract] OR "facebook"[Title/Abstract] OR "twitter"[Title/Abstract] OR "whatsapp"[Title/Abstract] OR "skyp"[Title/Abstract] OR "you-tube"[Title/Abstract] OR "google hangout"[Title/Abstract] OR "tiktok"[Title/Abstract] OR "email"[Title/Abstract] OR "electronic mail"[Title/Abstract] OR "text"[Title/Abstract] OR "multimedia"[Title/Abstract] OR "instant messenger"[Title/Abstract] OR "remind"[Title/Abstract] OR "message"[Title/Abstract] OR "sms"[Title/Abstract] OR "mms"[Title/Abstract] OR "voice call"[Title/Abstract] OR "callback"[Title/Abstract] OR "voice over internet"[Title/Abstract] OR "voip"[Title/Abstract] OR "virtual"[Title/Abstract] OR "virtual reality"[Title/Abstract] OR "augmented reality"[Title/Abstract] OR "artificial intelligence"[Title/Abstract] OR "AI"[Title/Abstract] OR "mobile app"[Title/Abstract] OR "computer-assisted technology"[Title/Abstract] OR "technology"[Title/Abstract] OR "computer"[Title/Abstract] OR "internet"[Title/Abstract] OR "technological invent"[Title/Abstract] OR "technological innovation"[Title/Abstract] OR "world wide web"[Title/Abstract] OR "webcast"[Title/Abstract] OR "digital"[Title/Abstract] OR "electronic"[Title/Abstract] OR "digital platform"[Title/Abstract] OR "digital technology"[Title/Abstract] OR "digital resource"[Title/Abstract] OR "digital intervention"[Title/Abstract] OR "web based digital health"[Title/Abstract] OR "web-based intervention"[Title/Abstract] OR "interactive technology"[Title/Abstract] OR "software"[Title/Abstract] OR "wireless"[Title/Abstract] OR "blue-tooth"[Title/Abstract] OR "tele-communication"[Title/Abstract] OR "social media"[Title/Abstract] 2275584  32. #19 or #20 or #21 or #22 or #23 or #24 or #25 or #26 or #27 or #28 or #29 or #30 or #31 2457259  33. "Social Networking"[Mesh] 6692  34. "Social Support"[Mesh] 85387  35. "Friends"[Mesh] 7332  36. "Family"[Mesh] 390144  37. "Peer Group"[Majr] 11562  38. "Social Media"[Mesh] 19638  39. "social network"[Title/Abstract] OR "social support"[Title/Abstract] OR "partner"[Title/Abstract] OR "friend"[Title/Abstract] OR "family"[Title/Abstract] OR "relative"[Title/Abstract] OR "spouse"[Title/Abstract] OR "wife"[Title/Abstract] OR "husband"[Title/Abstract] OR "support"[Title/Abstract] OR "peer"[Title/Abstract] OR "social care"[Title/Abstract] OR "perceived social support"[Title/Abstract] OR "online social"[Title/Abstract] OR "online social support"[Title/Abstract] OR "social citizen"[Title/Abstract] OR "social engag"[Title/Abstract] OR "social participation"[Title/Abstract] OR "husband-wife communication"[Title/Abstract] OR "partner communication"[Title/Abstract] OR "social relation"[Title/Abstract] OR "interpersonal relation"[Title/Abstract] OR "social tie"[Title/Abstract] OR "social integration"[Title/Abstract] OR "social bond"[Title/Abstract] OR "interpersonal connect"[Title/Abstract] OR "community connect"[Title/Abstract] OR "peer support"[Title/Abstract] OR "emotional support"[Title/Abstract] OR "relational close"[Title/Abstract] OR "belonging"[Title/Abstract] OR "social inclusion"[Title/Abstract] OR "connect"[Title/Abstract] OR "social connect"[Title/Abstract] OR "social interact"[Title/Abstract] 3709519  40. #33 or #34 or #35 or #36 or #37 or #38 or #39 3972968  41. social infrastruct*[Title/Abstract] OR social capital*[Title/Abstract] OR social trust*[Title/Abstract] OR civic engag*[Title/Abstract] OR social cohes*[Title/Abstract] OR interpersonal trust*[Title/Abstract] OR trust*[Title/Abstract] OR community participat*[Title/Abstract] OR reciprocit*[Title/Abstract] OR collective efficac*[Title/Abstract] OR disengag*[Title/Abstract] OR lonel*[Title/Abstract] OR social cohes*[Title/Abstract] OR social isolat*[Title/Abstract] 143065  42. "Internet-Based Intervention"[Mesh] 1681  43. Intervention*[Title/Abstract] OR program*[Title/Abstract] 2600141  44. #42 or #43 2600343  45. #40 and #44 577983  46. #3 and #18 and #32 and #45 1743 |
| --- |

**Table S2:** Quality assessment of included studies (cross-sectional studies) based on JBI criteria.

| **Author** | **Q1** | **Q2** | **Q3** | **Q4** | **Q5** | **Q6** | **Q7** | **Q8** | **Q9** | **Quality** |
| --- | --- | --- | --- | --- | --- | --- | --- | --- | --- | --- |
| Martinez et al. (2025) [1] | + | + | + | + | + | + | + | + | + | High |
| Pan et al. (2021) [2] | + | + | + | - | - | + | + | - | + | Moderate |
| Kouvonen et al. (2021) [3] | + | + | + | + | + | + | + | + | + | High |
| Jun, Galambos and Lee (2021) [4] | + | + | + | + | + | + | + | + | + | High |

Note: + yes; - no

Q1: Were the criteria for inclusion in the sample clearly defined?

Q2: Were the study subjects and the setting described in detail?

Q3: Was the exposure measured in a valid way?

Q4: Was the exposure measured in a reliable way?

Q5: Were objective, standard criteria used for measurement of the condition?

Q6: Were confounding factors identified?

Q7: Were strategies to deal with confounding factors stated?

Q8: Were the outcomes measured in a valid and reliable way?

Q9: Was appropriate statistical analysis used?

**Table S3:** Quality assessment of included studies (qualitative studies) based on JBI criteria.

| **Author** | **Q1** | **Q2** | **Q3** | **Q4** | **Q5** | **Q6** | **Q7** | **Q8** | **Q9** | **Q10** | **Quality** |
| --- | --- | --- | --- | --- | --- | --- | --- | --- | --- | --- | --- |
| Adeniji and Ashirifi (2024) [5] | + | + | + | + | + | + | + | + | + | + | High |
| Juul, Wilding and Baldassar (2019) [6] | + | + | + | + | + | - | + | + | + | + | High |
| Khvorostianov, Elias and Nimrod (2012) [7] | - | + | + | + | + | + | + | + | - | + | Moderate |
| Millard, Baldassar and Wilding (2018) [8] | + | + | + | + | + | + | + | + | + | + | High |
| Nguyen, Baldassar and Wilding (2022) [9] | + | + | + | + | + | + | + | + | + | + | High |

Note: + yes; - no

Q1: Is there congruity between the stated philosophical perspective and the research methodology?

Q2: Is there congruity between the research methodology and the research question or objectives?

Q3: Is there congruity between the research methodology and the methods used to collect data?

Q4: Is there congruity between the research methodology and the representation and analysis of data?

Q5: Is there congruity between the research methodology and the interpretation of results?

Q6: Is there a statement locating the researcher culturally or theoretically?

Q7: Is the influence of the researcher on the research, and vice- versa, addressed?

Q8: Are participants, and their voices, adequately represented?

Q9: Is the research ethical according to current criteria or, for recent studies, and is there evidence of ethical approval by an appropriate body?

Q10: Do the conclusions drawn in the research report flow from the analysis, or interpretation, of the data?

**References**

1. DeLange Martinez P, Tancredi D, Pavel M, Garcia L, Young HM. Adapting the technology acceptance model to examine the use of information communication technologies and loneliness among low-income, older Asian Americans: cross-sectional survey analysis. JMIR Aging. Jan 8, 2025;8:e63856.

2. Pan H, Fokkema T, Switsers L, Dury S, Hoens S, De Donder L. Older Chinese migrants in coronavirus pandemic: exploring risk and protective factors to increased loneliness. Eur J Ageing. Jun 2021;18(2):207-215.

3. Kouvonen A, Kemppainen L, Ketonen EL, Kemppainen T, Olakivi A, Wrede S. Digital information technology use, self-rated health, and depression: population-based analysis of a survey study on older migrants. J Med Internet Res. Jun 14, 2021;23(6):e20988.

4. Jun JS, Galambos C, Lee KH. Information and communication technology use, social support, and life satisfaction among Korean immigrant elders. J Soc Serv Res. Jul 4, 2021;47(4):537-552.

5. Adeniji DO, Ashirifi GD. The voices of older African immigrants on how they cope with social isolation and loneliness in the United States. Clin Gerontol. Mar 15, 2025;48(2):260-269.

6. Juul A, Wilding R, Baldassar L. The best day of the week: new technology enhancing quality of life in a care home. Int J Environ Res Public Health. Mar 19, 2019;16(6):1000-1016.

7. Khvorostianov N, Elias N, Nimrod G. ‘Without it I am nothing’: the internet in the lives of older immigrants. New Media & Society. Jun 2012;14(4):583-599.

8. Millard A, Baldassar L, Wilding R. The significance of digital citizenship in the well-being of older migrants. Public Health (Fairfax). May 2018;158:144-148.

9. Nguyen HT, Baldassar L, Wilding R. Lifecourse transitions: how ICTS support older migrants’ adaptation to transnational lives. Social Inclusion. 2022;10(4):181-193.
